# Thyrotropin Regulates eNOS Expression in the Endothelium by PGRN Through Akt Pathway

**DOI:** 10.3389/fendo.2018.00353

**Published:** 2018-07-05

**Authors:** Fengwei Jiang, Haoyu Wang, Suqing Bao, Haicheng Zhou, Yuanyuan Zhang, Yumeng Yan, Yaxin Lai, Weiping Teng, Zhongyan Shan

**Affiliations:** ^1^Department of Endocrinology and Metabolism, Institute of Endocrinology of the First Affiliated Hospital China Medical University, Shenyang, China; ^2^The First Affiliated Hospital of Jinzhou Medical University, Jinzhou, China

**Keywords:** subclinical hypothyroidism, thyrotropin, endothelial nitric oxide synthase, progranulin, human umbilical vein endothelial cells, Akt

## Abstract

To investigate the expression of endothelial nitric oxide synthase (eNOS) and nitric oxide (NO) in the aorta of subclinical hypothyroidism (SCH) rat model. The mechanisms underlying thyrotropin (TSH) affecting eNOS and PGRN expression in human umbilical vein endothelial cells (HUVECs) cultured *in vitro* were investigated. In the current study, SCH rat models were established by the administration of L-T_4_ injection after thyroidectomy in Wistar rats, as opposed to that in the normal and clinical hypothyroidism (CH) groups. The concentrations of NO (pmol/μL) in the SCH and CH groups were significantly lower than that in the normal group (40.8 ± 7.6 and 32.9 ± 10.8 vs. 51.2 ± 12.1, *P* < 0.05). However, the expression level of eNOS is increased significantly (*P* < 0.05) in both SCH and CH groups; a similar result was observed for the PGRN protein. In cultured HUVECs, TSH can also up-regulate the expression of eNOS; however, it is accompanied by a reduced concentration of NO and increased level of superoxide anion, thereby indicating uncoupled eNOS. As eNOS is increased, we found that Akt in HUVECs were upregulated by TSH, as well as PGRN expression. While inhibiting the expression of PGRN in HUVECs using siRNA, the expression of eNOS, as well as Akt were also inhibited. In conclusion, SCH can induce vascular endothelial dysfunction in rats, and PGRN participated in the process of TSH-induced expression of Akt/eNOS in the endothelium.

## Introduction

Endothelial dysfunction is the early stage of many diseases, such as hypertension and atherosclerosis (1, 2). The vascular endothelium expressed the receptor of TSH; however, its function was not clear (3). Endothelial nitric oxide synthase (eNOS) is a crucial enzyme in the production of nitric oxide (NO) generation and plays a vital role in anti-atherosclerosis (4).

Reportedly, although clinical hypothyroidism reduces the expression of eNOS in vascular endothelium, it is increased in the cardiac tissue (5). Virdis et al. demonstrated that eNOS was not altered in methimazole-induced hypothyroidism rat (6). Other groups studied the endothelial function including macrovasculatures and resistance vessels in hypothyroidism induced by propylthiouracil. Subclinical hypothyroidism (SCH) is an independent risk factor of atherosclerosis; however, its influence on vascular endothelial function is yet controversial (7, 8). This might be attributed to the lack of SCH animal models, and only indirect indicators can be obtained in the human study such as serum indicators or invasive examinations (9–15). Some findings suggested that SCH can induce vascular endothelial dysfunction; nevertheless, the underlying mechanisms are yet elusive (9–13).

Progranulin (PGRN), a growth factor with many functions, is widely distributed in different cells. PGRN exerts various functions in cell growth, traumatic healing, and tumor formation. In recent years, its role in inflammation has been studied intensively (16–18). In 2009, Kojima et al. first reported that PGRN expressed in atherosclerosis plaque (19). Another study found that PGRN had predictive value on carotid intima-media thickness (subclinical atherosclerosis stage), independent of other cardiovascular risk factors (20). Furthermore, PGRN was reported to upregulate the Akt/eNOS phosphorylation level in HUVECs (21). Although only a few studies have addressed the PGRN and vascular endothelial function, the studies on the role of PGRN in the effect of TSH on vascular endothelial function are absent.

This study investigated the influence of TSH on vascular eNOS and its relationship with PGRN in SCH rat models and HUVECs cultured *in vitro* to understand the mechanism underlying TSH-induced endothelial dysfunction.

## Materials and methods

### Animals

Thirty male Wistar rats, weighing 180–200 g, were acquired from Vital River Laboratory Animal Technology Co. (Beijing, China) at the age of 6 weeks (6 w). All animals were randomly divided into 3 groups: normal control (NC, *n* = 10), subclinical hypothyroidism (SCH, *n* = 10), and clinical hypothyroidism (CH, *n* = 10). Rats in the CH and SCH groups underwent total thyroidectomy, while the NC group received sham surgery without the removal of the thyroid gland. After 4 w, the rats in the SCH group were administered a subcutaneous injection of L-T_4_ (s.c 1.0 μg/100 g, Sigma, St. Louis, USA) daily for 14 w. The NC and CH groups were administered physiological saline injection. Serum TSH and total T_4_ levels were detected every 4 w post-surgery (Immulite, Diagnostic Products Co., Los Angeles, USA). All rats were sacrificed 14 w after drug injection. Serum samples and aortas were collected and stored at −80°C.

The inter-assay coefficients of variation for TSH and TT_4_ were 1.73–5.75% and 1.26–3.27%, respectively. The intra-assay coefficients of variation for TSH and TT4 were 1.16–4.12 and 4.34–6.13%, respectively. The animal experiments were approved by the Animal Care and Use Committee of the First Affiliated Hospital of China Medical University and adhered to the guidelines of the National Institute of Health Guide for animals.

### Cell culture

Human umbilical vein endothelial cells (HUVECs, ScienCell, Carlsbad, USA) were cultured in endothelial cell medium (ECM, ScienCell, Carlsbad, USA) in a humidified incubator (37°C, 5% CO_2_). HUVECs were stimulated by TSH after starvation for 12 h.

Inhibition of PGRN expression using siRNA: HUVECs were changed to opti-MEM serum-free medium (Gibco, USA) after washing twice with phosphate-buffered saline (PBS). Then, Lipofectamine^TM^2000/siRNA compound (60 pmol siRNA/well) was added. Subsequently, the transfection complex with normal ECM was removed after 6 h culture in the incubator.

MK-2206 (Selleck, USA), an inhibitor of Akt, was dissolved in DMSO (the final concentration of DMSO was less than 0.1%). HUVECs were pretreated with MK-2206 (1 μM) when evaluating the AKT pathway.

### Detection of NO concentrations

The aortic tissues homogenates were centrifuged at 12,000 rpm and 4°C for 15 min. The supernatants were extracted to detect the NO concentrations in aortic tissues (Nitric Oxide Fluorometric Assay Kit, BioVision, USA).

HUVECs, inoculated in a 96-well plate, were stimulated with different concentrations of TSH for 24 h. The ECM was removed, and diluted DAF-FM DA (3-amino, 4-aminomethyl-2′, 7′-difluorescein, diacetate) added (5 μmol/L, 200 μL/well, Beyotime, China), followed by incubation for 20 min. After washing for 3 times, fluorescence was detected by the microplate reader (excitation = 495 nm and emission = 515 nm) adjusted by the protein concentration in each well.

### Detection of serum PGRN concentrations

Serum samples of all the rats were obtained and stored at −80°C. The PGRN concentrations in the serum were detected using Rat PGRN ELISA Kit (Horabio, Shanghai, China).

### Detection of superoxide anion in cell supernatants

HUVECs were diluted with various concentrations of TSH, and cell supernatants were collected. Colorimetric method was used to determine the level of superoxide anion in cell supernatants.

### Western blot

BCA method was used to detect the concentrations of protein extracted from the aorta of rats or HUVECs. The proteins were separated by SDS-PAGE and transferred to PVDF membranes. The membranes were blocked with 0.05% TBST containing 5% BSA for 1 h, followed by probing with the following primary antibodies at 4°C overnight: anti-eNOS, anti-p-eNOS (S1177) (1:500, Cell Signaling Technology, USA); anti-Akt, anti-p-Akt (Ser473) (1:1,000, Abcam, USA); anti-PGRN (1:1,000, Santa Cruz, USA). Subsequently, the membranes were incubated for 1 h with peroxidase-conjugated secondary antibody (1:3,000, Zhongshan Golden Bridge Biotechnology Co. Ltd., China). The immunoreactive bands were detected by chemiluminescence.

### Real-time PCR

Total RNA was extracted using TRIzol and reverse-transcribed to cDNA using a PrimeScript^TM^ RT Kit. The samples were mixed with SYBR® Premix ExTaq^TM^, primers, and DEPC water. The primer pairs were as follows: *eNOS*, 5′-CTC GTC CCT GTG GAA AGA CAA-3′ (forward) and 5′-TGA CTT TGG CTA GCT AGC TGG TAA CTG T-3′ (reverse); *PGRN*, 5′-TGT GTA GCT GAG GGG CAG TGT-3′ (forward) and 5′-GAT GTC TCT GGG GTG GGA TAA G-3′ (reverse); *GAPDH*, 5′-CAA TGA CCC CTT CAT TGA CC-3′ (forward) and 5′-CTC GTC CCT GTG GAA AGA CAA-3′ (reverse). The reagents were purchased from Takara (Shiga, Japan). The reactions were performed on LightCycler Real-Time PCR System (Roche 480, Berlin, Germany).

## Statistical analysis

SPSS 16.0 software was used for analysis. Data were represented as mean ± SEM. The one-way ANOVA analysis was used for multiple comparisons. Data were obtained for a minimum of three experiments. Statistical significance was considered at *P* < 0.05.

## Results

### Thyroid function in different rat models

Serum TSH and TT_4_ were monitored in all the rats of NC, SCH, and CH groups. Increased TSH and normal serum TT_4_ levels in the SCH group as compared to the NC group 4 w after L-T_4_ injection to 14 w indicated the successful establishment of the SCH rat model. Consecutively, increased TSH and decreased TT_4_ levels were also observed in the CH group (22).

### Expression of NO and eNOS in different rats

Fluorometric method was used to detect the concentrations of NO in aortic tissues. The NO levels (pmol/μL) in the SCH and CH groups were less than that in the NC group (40.8 ± 7.6 and 32.9 ± 10.8 vs. 51.2 ± 12.1, *P* < 0.05).

**Figure 1:**
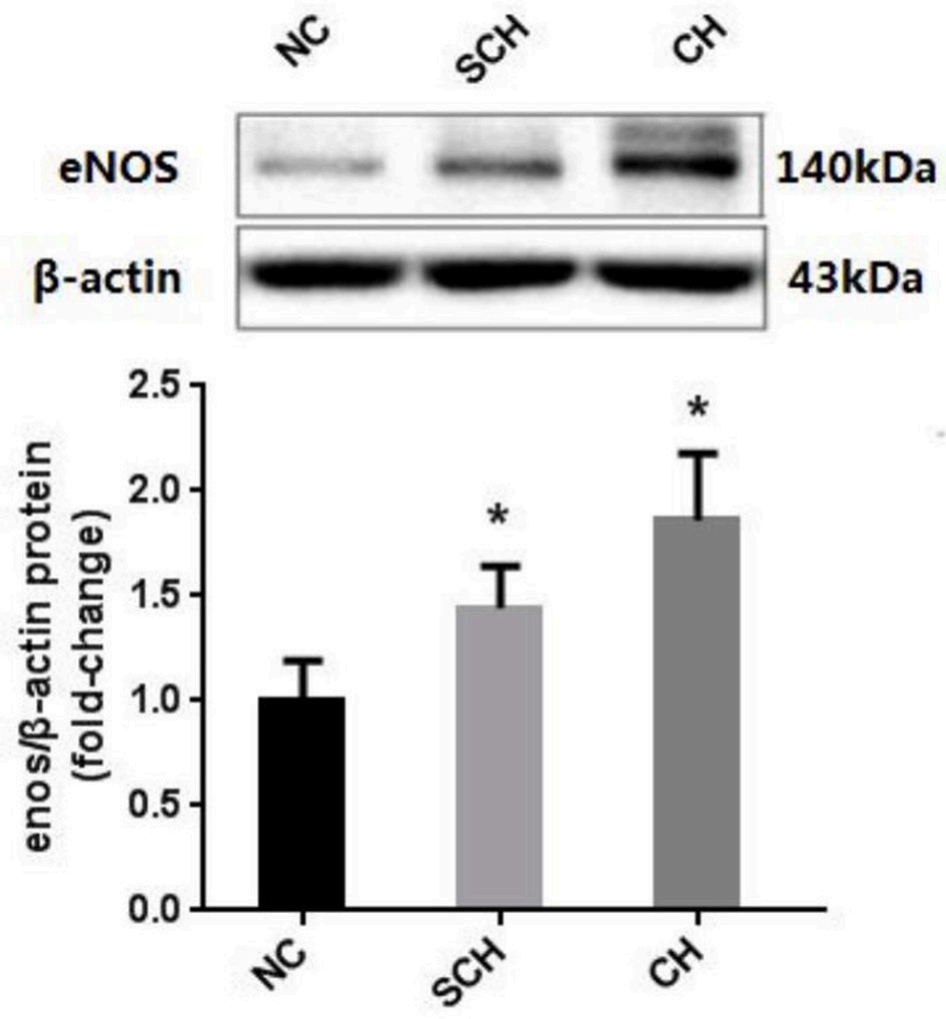
Expressions of eNOS in the aorta of the three groups. Protein samples were extracted from the aorta in rats of NC, SCH, and CH groups. *N* = 10 in each group. Error bars represent mean ± SEM; ^*^P < 0.05, when compared with the NC group.

Moreover, Western blot showed that the protein expressions of aortic eNOS in the CH and SCH groups were significantly increased than the NC group (*P* < 0.05) (Figure 1).

### Expression of PGRN in rats of different groups

**Figure 2:**
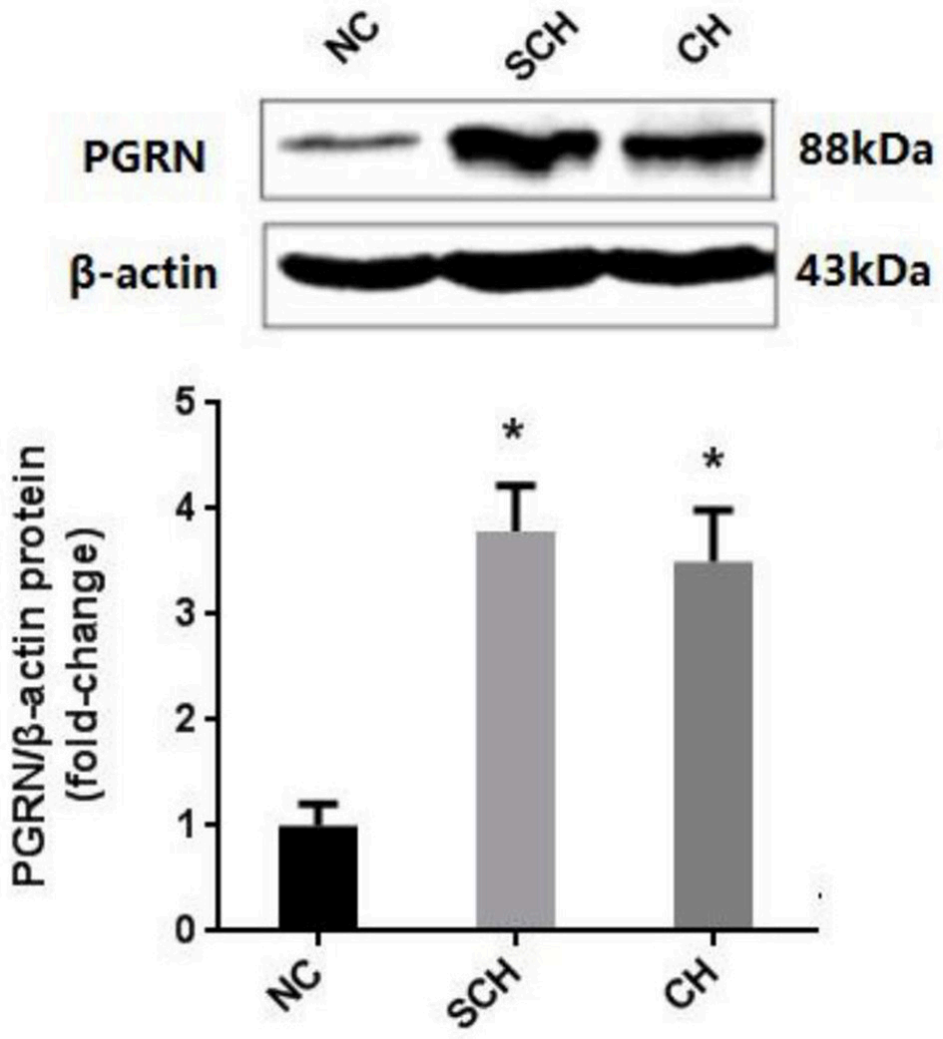
Protein expression of PGRN in aortic tissue in different groups. NC, normal control; SCH, subclinical hypothyroidism; CH, clinical hypothyroidism; *N* = 10 in each group. ^*^P < 0.05, when compared with the NC group.

Compared to the NC group, the PGRN expressions in aorta were significantly increased in the SCH and CH groups as evaluated by Western blot (*P* < 0.05) (Figure 2).

The serum concentrations of PGRN in the SCH group (42.0 ± 7.3 ng/ml) and CH group (39.6 ± 3.1 ng/ml) were significantly higher than that in the NC group (30.7 ± 8.5 ng/ml) (*P* < 0.05), which was in accordance with the results of Western blot. However, no significant difference was observed between the SCH and CH groups.

### Effect of TSH on the expression of eNOS/NO in HUVECs *in vitro*

**Figure 3:**
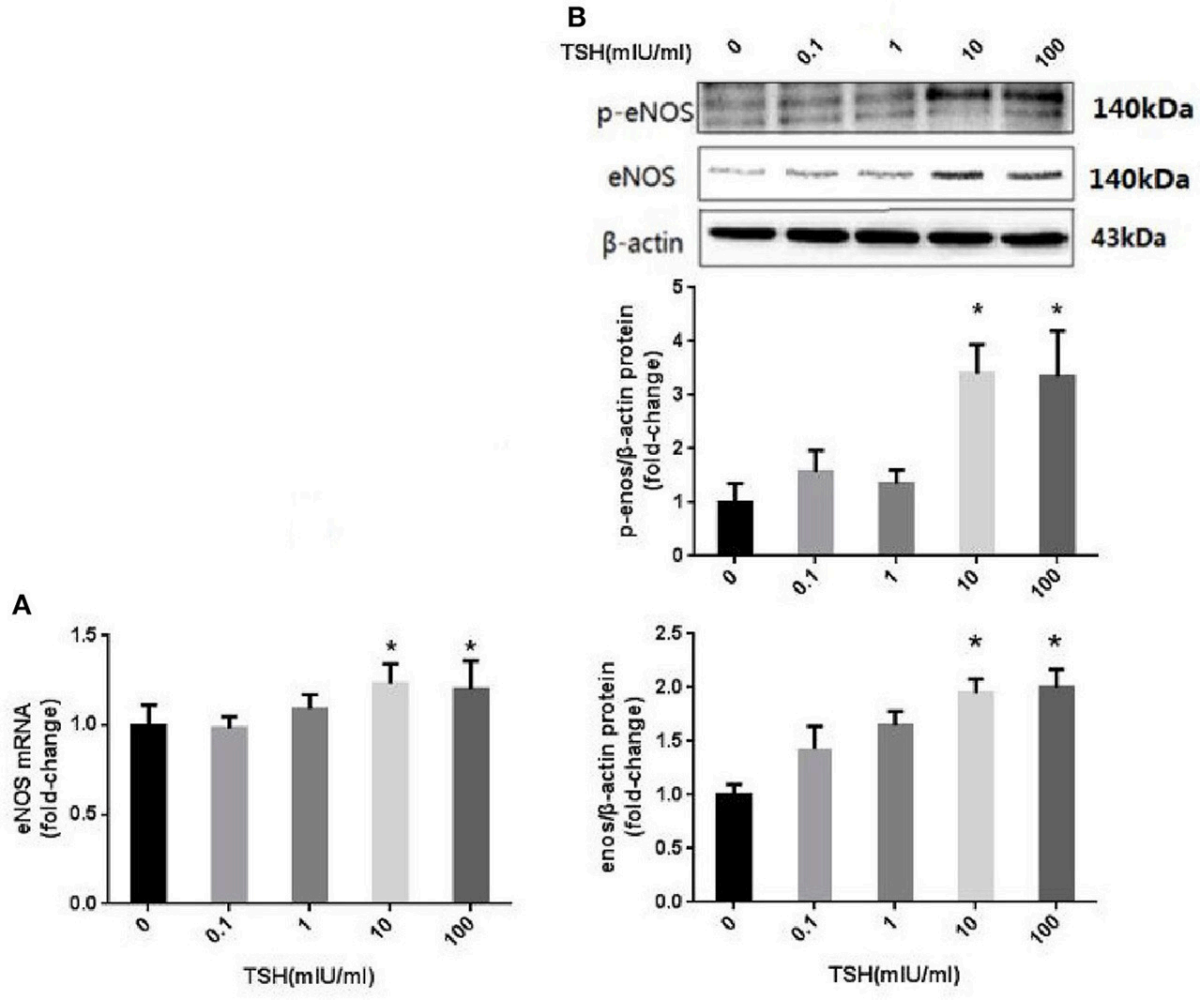
eNOS expression and its phosphorylation in HUVECs stimulated by different concentrations of TSH. **(A)** mRNA expression of eNOS in HUVECs stimulated by different concentrations of TSH (0, 0.1, 1, 10, and 100 mIU/ml); **(B)** Protein expression of eNOS in HUVECs treated with different concentrations of TSH. Data were obtained from three separate experiments. HUVECs without stimulating with TSH (0 mIU/ml) served as control; ^*^P < 0.05, when compared with the control.

HUVECs were stimulated with different concentrations of TSH (0, 0.1, 1, 10, and 100 mIU/ml) for 24 h. The protein and mRNA expressions in HUVECs stimulated by 10 and 100 mIU/ml TSH were up-regulated significantly (*P* < 0.05) as compared to no stimulation with TSH, and the phosphorylation of eNOS (S1177) was also increased (Figure 3).

**Figure 4:**
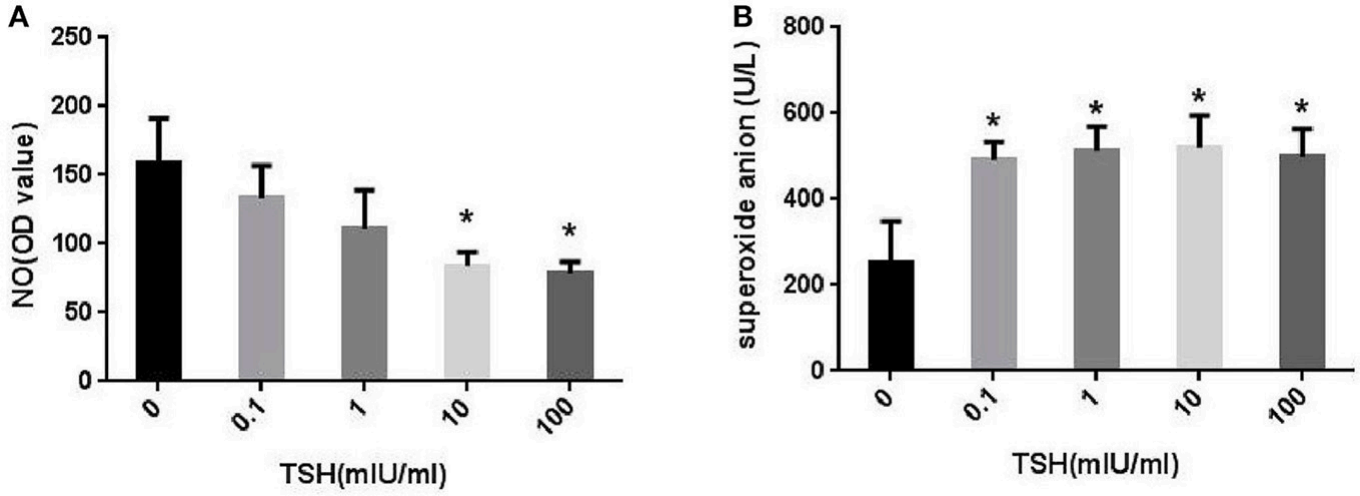
Effect of TSH on NO and superoxide anion levels *in vitro*. HUVECs were stimulated by different concentrations of TSH (0, 0.1, 1, 10, and 100 mIU/ml) for 24 h. **(A)** NO levels in HUVECS and the OD values of NO were adjusted by concentrations of protein extracted from HUVECs; **(B)** Superoxide anion levels in the supernatant; Data were obtained from three separate experiments. ^*^P < 0.05, when compared to the control.

However, the levels of NO in the cells decreased gradually as the concentrations of TSH increased. When the concentrations of TSH increased to 10 and 100 mIU/ml, a significant difference was observed as compared to the control (Figure 4A). Consecutively, we also collected the supernatant to detect the levels of superoxide anion. The results showed that TSH stimulation could significantly increase the levels of superoxide anion, even in the minimum concentration (Figure 4B). This phenomenon suggested that eNOS was uncoupled.

### Effect of Akt and PGRN in the up-regulation of eNOS induced by TSH

**Figure 5:**
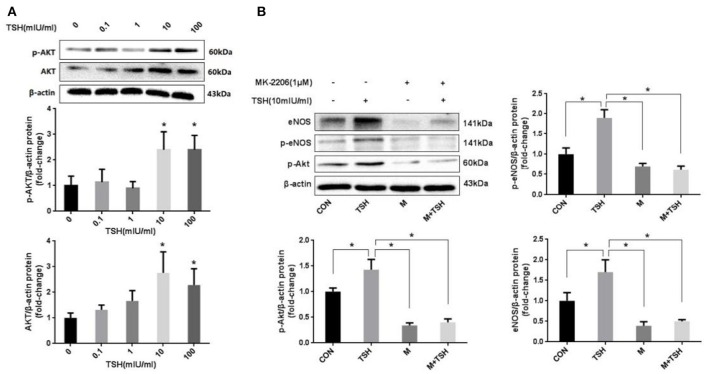
Role of Akt in the induction by TSH of eNOS in HUVECs. **(A)** Protein expression of Akt in HUVECs stimulated by different concentrations of TSH (0, 0.1, 1, 10, and 100 mIU/ml) for 24 h; **(B)** Inhibition of Akt: HUVECs were pretreated with 1 μM of MK-2206 for 6 h or not, and then together with or without TSH (10 mIU/ml) for 24 h. Data were obtained from three separate experiments. HUVECs without stimulating with TSH (0 mIU/ml) served as control; M represented MK-2206; ^*^P < 0.05, when compared with the control.

Akt is an critical signaling pathway regulating eNOS in the endothelium. The present study found that up-regulation of TSH-induced eNOS was accompanied by an increased expression of Akt and its phosphorylation (Figure 5A). This phenomenon suggested that Akt might participate in the regulation of TSH on the expression of eNOS. MK-2206 was used to inhibit the activity of Akt. The results showed that it attenuated the TSH-induced phosphorylation of Akt. In addition, MK-2206 can block TSH induction of eNOS (Figure 5B).

**Figure 6:**
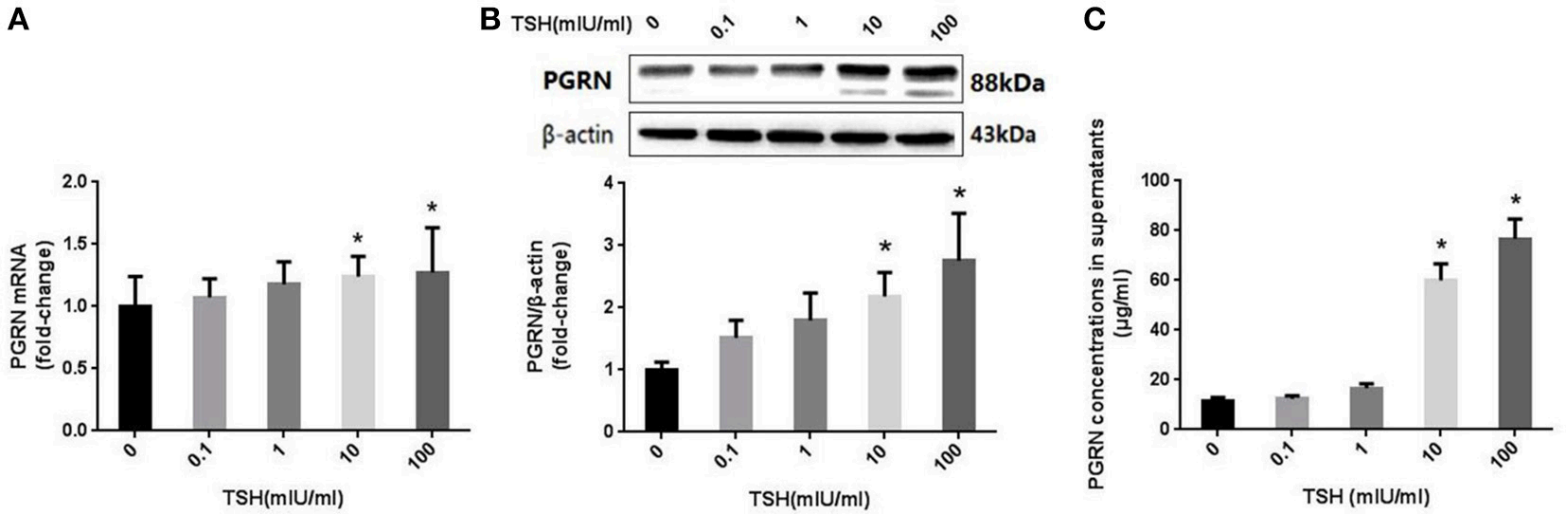
Effect of TSH on PGRN in HUVECs. HUVECs were stimulated by different concentrations of TSH (0, 0.1, 1, 10, and 100 mIU/mL) for 24 h. **(A)** mRNA level of PGRN in HUVECs stimulated by different concentrations of TSH as assessed by real-time PCR; **(B)** Protein expression of PGRN in HUVECs treated with different concentrations of TSH as assessed by Western blot; this actin from figure 3 was re-used here, because PGRN was detected at the same time with eNOS in one experiment of WB; **(C)** PGRN concentrations in supernatant from HUVECs stimulated by different concentrations of TSH. Data were obtained from three separate experiments. HUVECs without stimulating with TSH (0 mIU/mL) served as control; ^*^P < 0.05, when compared with the control.

We detected the expression of PGRN using different methods in HUVECs treated with different concentrations of TSH (0, 0.1, 1, 10, and 100 mIU/ml) for 24 h. The results showed that both mRNA and protein expressions of PGRN were increased significantly in HUVECs stimulated with 10 and 100 mIU/ml TSH (*P* < 0.05), similar to the changes of eNOS. In addition, the levels of PGRN in the supernatant were also increased (Figure 6).

**Figure 7:**
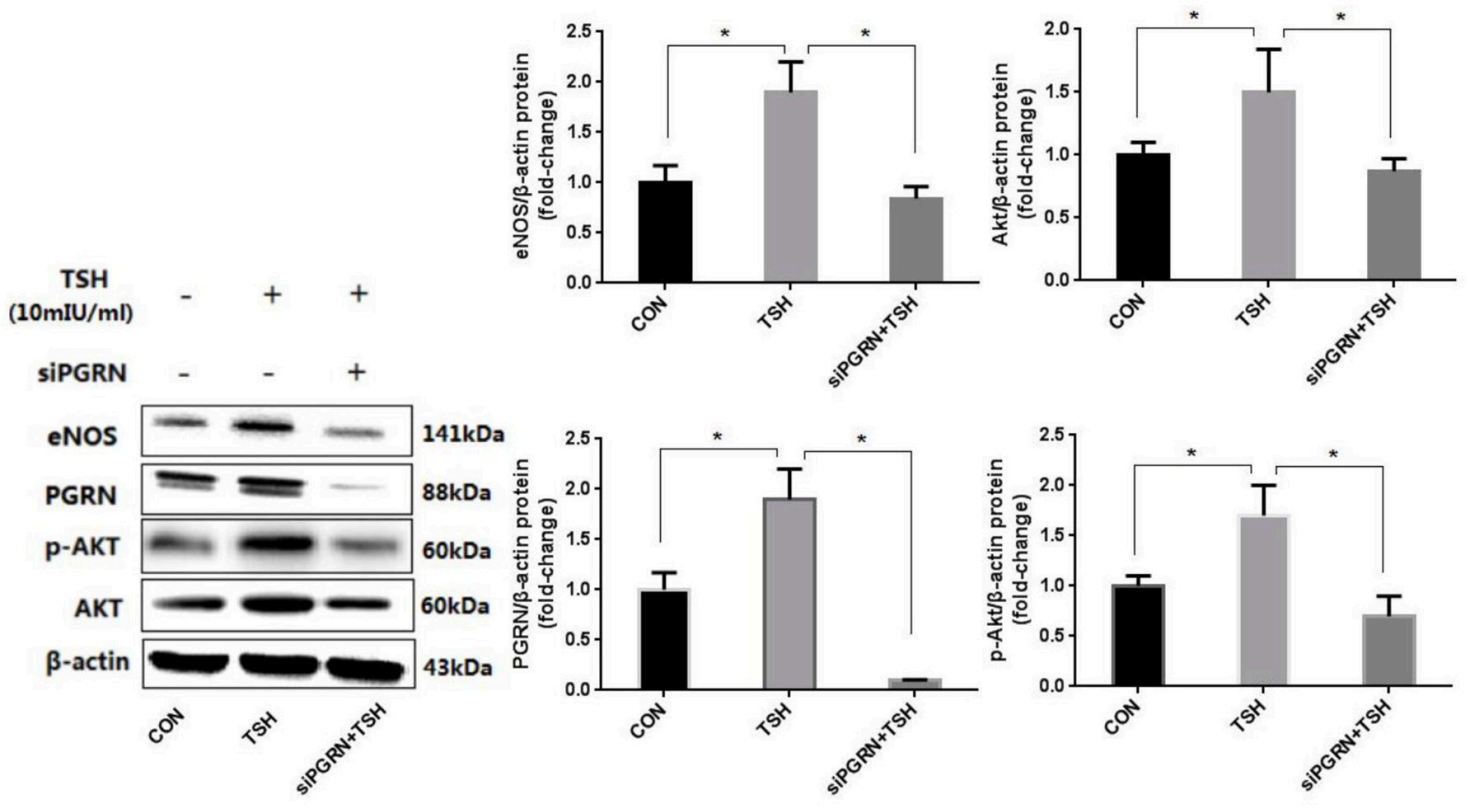
PGRN inhibition can prevent eNOS upregulation induced by TSH in HUVECs. siPGRN was used to inhibit the expression of PGRN in HUVECs; the concentration of TSH was 10 mIU/ml, stimulating for 24 h; Data were obtained from three separate experiments. ^*^P < 0.05, when compared with the control.

The above findings showed that the change in PGRN were in agreement with the altered eNOS. In order to further investigate the relationship between them, we inhibited expression of PGRN in HUVECs using siRNA before TSH stimulation. Subsequently, the expression of eNOS was neither upregulated by TSH nor Akt and its phosphorylation (Figure 7).

## Discussion

The present study found that the expression of the aortic endothelial eNOS and PGRN increased in SCH rats; however, it decreased with respect to the NO concentration in the aorta. In the HUVECs cultured *in vitro*, TSH can increase eNOS expression through the Akt pathway, relating to increased PGRN. Although the eNOS expression was up-regulated, increased superoxide anion and reduced NO level indicated eNOS uncoupling.

The decline in the physiological activity of endothelial NO is a major indicator of vascular endothelial dysfunction and a crucial mechanism for atherosclerosis. Clinical studies have found that some patients with SCH were accompanied by decreased endothelial-dependent vasodilation function (FMD, reflecting the vascular endothelial NO ability), and TSH level was negatively related to the FMD, which can be improved by L-T4 replacement (23, 24). The current study displayed that decreased NO in the rat aorta existed not only in clinical hypothyroidism but also in SCH. Although only TSH was altered, SCH can induce vascular endothelial dysfunction.

Our results showed that TSH stimulation increased the expression of eNOS in HUVECs. eNOS is known as a double-edged sword in cardiovascular system: NO is produced from the substrate, L-arginine, under the effect of eNOS in physiological condition playing the role of protection; in the pathological state, decreased level of NO and increased level of superoxide anion can generate ONOO^−^, thereby aggravating the oxidative stress reaction that is termed as eNOS uncoupling (25–27). eNOS uncoupling commonly exists in many diseases, such as hypertension and atherosclerosis. The mechanisms of eNOS uncoupling are complex, encompassing L-arginine deficiency, increased concentration of ADMA (an endogenous nitric oxide synthase inhibitor), lack of cofactor of four hydrogen biopterin, and oxidative stress (28, 29). Balzan et al. found that TSH promoted angiogenesis in human dermal microvascular endothelial cells through the cAMP-mTOR pathway; simultaneously, the levels of vascular endothelial growth factor (VEGF) and eNOS were increased (30). This effect is also present in endothelial cells of the human cardiac microvascular and rat aorta (30). However, another study showed that TSH reduces the expression of eNOS in cultured HUVECs (31). In our study, although TSH up-regulated the expression of eNOS, the concentration of NO decreased and the superoxide anion increased, indicating that TSH induced eNOS uncoupling (32). Previous studies also demonstrated that the increase in eNOS due to increased peroxide was a characteristic pathological change in atherosclerosis (33).

The current study investigated the expression of PGRN in subclinical and clinical hypothroidism rats for the first time. The expression of PGRN increased in serum and aorta in subclinical and clinical hypothyroidism rats. The TSH-stimulated increase in PGRN led to the up-regulation of eNOS through Akt signaling pathway in HUVECs *in vitro*. Akt exerts an important role in mediating TSH actions in thyroid. This pathway was also very important in TSH mediating eNOS. Atherosclerosis is known to occur as a series of inflammations depending on the endothelial injury (2). PGRN is a pivotal inflammatory modulator, highly expressed in inflammation and injury. It can directly bind to TNF receptors and disrupt the TNF-α-mediated responses (20). Conversely, PGRN exerted the pro-inflammatory functions in high-fat diet-induced insulin-resistance and obesity. These results suggested that PGRN may exert dual functions in inflammation (18). PGRN was highly expressed in foam cells and macrophages of atherosclerotic plaques, and knockout of PGRN resulted in severe atherosclerotic lesions (34). Previous results showed that serum PGRN was closely related to inflammatory factors and considered as an independent predictor of atherosclerosis in patients with metabolic syndrome. Furthermore, PGRN influences the early stage of atherosclerosis, and the mechanisms might be correlated with the inflammatory factors instead of conventional cardiovascular risk factors (20). Kojima et al. reported that PGRN inhibits the MCP-1-mediated monocyte migration in atherosclerosis, as well as, enhance the TNF-α-mediated migration of aortic smooth muscle cells (19). Owing to the complex role of PGRN, its effects in TSH-induced vascular endothelial dysfunction necessitate further studies.

The deficiencies in this study include lack of morphological study of the aortic endothelial microstructural changes in subclinical hypothyroidism rats. The mechanism underlying eNOS uncoupling is complicated, thereby demanding further studies. Akt phosphorylation has not been studied in the aortas for technical reasons: it is hard to separate only the endothelial cells from the aortas for experiments and quantity of endothelial cells in aortas was limited.

**Figure 8:**
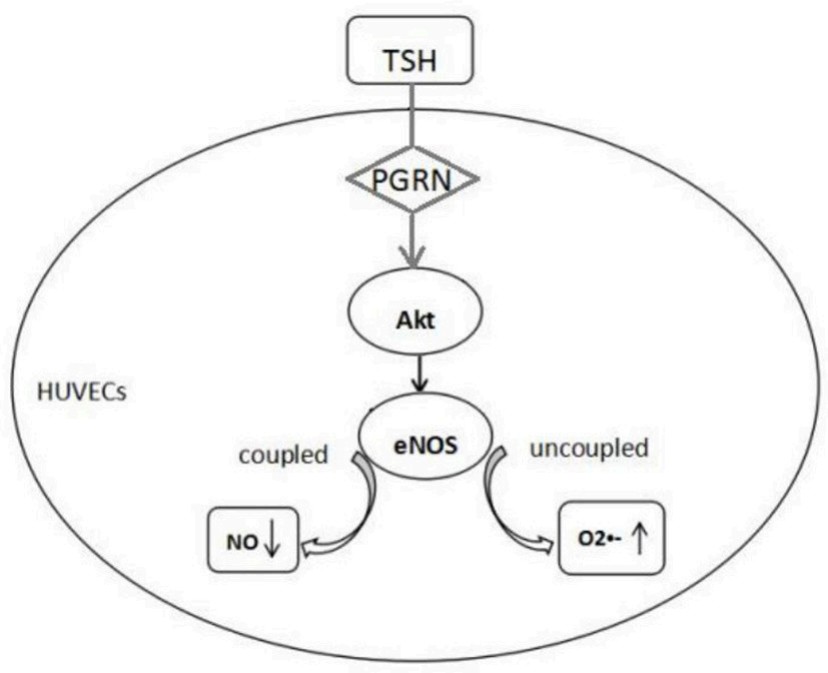
Proposed mechanism of endothelial dysfunction induced by TSH. TSH up-regulated eNOS expression in HUVECs by PGRN through Akt pathway. Reduction in NO production and increase in superoxide anion indicated uncoupled eNOS.

In conclusion, endothelial dysfunction existed in the SCH, PGRN participated in the process of TSH up-regulating the eNOS expression in the endothelium through Akt pathway. However, TSH reduced the production of NO indicating uncoupled eNOS, which resulted in endothelial dysfunction (Figure 8).

## Author contributions

FJ was the first author and completed most of the research with the help of other authors. HW did many work in animal experiment. ZS was corresponding author and gave guidance for the research as well as WT.

### Conflict of interest statement

The authors declare that the research was conducted in the absence of any commercial or financial relationships that could be construed as a potential conflict of interest.
